# P02-01 Promoting Physical Activity in Secondary School for Health, a collaborative European project

**DOI:** 10.1093/eurpub/ckac095.020

**Published:** 2022-08-29

**Authors:** Léna Lhuisset, Julien Bois, Nicolas Fabre, Gautier Zunquin, Caera Grady, Leen Haerens, Jorge Mota, Javier Zaragoza, Lionel Dubertrand

**Affiliations:** 1 e2s UPPA, MEPS, Université de Pau et des Pays de l'Adour, Tarbes, France; 2 Department of Physical Education and Sport Sciences, Faculty of Education and Health Science, University of Limerick, Limerick, Ireland; 3 Department of Movement & Sports Sciences -Sports Pedagogy, Ghent University - Faculty of Medicine and Health Sciences, Ghent, Belgium; 4 CIAFEL-Faculty of Sports, University of Porto, Porto, Portugal; 5 EFYPAF-Faculty of Human and Education Sciences, University of Zaragoza, Zaragoza, Spain; 6 Sport and health centre, City of Tarbes, TARBES, France

**Keywords:** Implementation, Evaluation, Tools, Secondary school, Co-design approach

## Abstract

**Issue:**

The 2PASS-4Health project (Promoting Physical Activity in Secondary School for Health) was founded by the Erasmus+ Sport programme of the European Union to improve the participation in sport and physical activity (PA). This project aims at examining examples of PA promotion interventions in secondary school, identifying good practices as well as the main barriers and difficulties linked to the design, implementation and evaluation of such interventions in order to improve their quality and sustainability. This project targets both the scientific community and various stakeholders involved in school-based PA promotion for adolescents to provide them with clear knowledge and usable tools. The overall design of this project will be presented as well as the first results and deliverables created to implement the interventions.

**Description:**

In order to fill the existing gap between theory and practice by identifying evidence-based practices that work we are translating recent scientific knowledge into accessible information and tools that meet the needs of the stakeholders in the field. Furthermore, using a co-design approach involving several internationally recognised experts, stakeholders, and end-users, we developed some adapted and ready-to-use contents and we designed an optimised multicomponent school-based intervention that has been implemented in France and Spain. We evaluated these interventions not only in terms of outcomes related to PA and sedentary time, but also in relation to other important domains like implementation or maintenance based on the RE-AIM framework.

**Results:**

We are producing: (1) a white paper geared towards professionals and policy makers, and a consensus statement intended for the scientific community; (2) two handbooks on the implementation of the intervention and its evaluation, accompanied by two scientific publications; (3) an educational toolkit to support PA promotion in schools; and (4) articles on the evaluation of the interventions held in France and Spain.

**Lessons:**

System approach seems needed to implement a sustainable multilevel whole-of-school intervention co-constructed with the different stakeholders and end-users involved.

